# Effect of crosslinking/antioxidant agents as final irrigant on the fracture resistance of endodontically treated root after radiotherapy

**DOI:** 10.1371/journal.pone.0311132

**Published:** 2024-10-17

**Authors:** Maryam S. Tavangar, Fereshteh Shafiei, Sepehr Eslami Pirharati, Mohsen Bakhshandeh, Yasamin Ghahramani

**Affiliations:** 1 Department of Operative Dentistry, Oral and Dental Disease Research Center, School of Dentistry, Shiraz University of Medical Sciences, Shiraz, Iran; 2 Student Research Committee, Shiraz University of Medical Sciences, Shiraz, Iran; 3 Allied Medical Faculty, Radiology Technology Department, Shahid Beheshti University of Medical Sciences, Tehran, Iran; 4 Department of Endodontics, School of Dentistry, Shiraz University of Medical Sciences, Shiraz, Iran; University of Puthisastra, CAMBODIA

## Abstract

**Aim:**

To investigate the effects of Epigallocatechin gallate (EGCG) and curcumin, as a final irrigant on the fracture resistance of irradiated root that obturated with an epoxy resin sealer.

**Methodology:**

Eighty mandibular premolars were randomly divided into non-irradiated (NIR) and irradiated (IR) groups. The teeth were irradiated at 2 Gy per fraction, 5 times a week for a total dose of 60 Gy over 6 weeks. All specimens were decoronated, remaining 13±1 mm root length. Two groups were subdivided into four groups (n = 10): 1) non-instrumented; the intact root served as control. The other roots were instrumented with a pro-taper NiTi rotary system. The final irrigation used was 17% EDTA, followed by three irrigation solution groups; 2) 2.5% NaOCl, 3) 0.02% EGCG, and 4) 0.1% curcumin. Root canals were filled with gutta-percha and AH plus. All specimens were embedded in self-curing acrylic resin and loaded vertically at 1 mm/min until fracture occurred. Also, sealer penetration was assessed by confocal laser scanning microscopy (CLSM). The data were evaluated statistically using two-way ANOVA and Tukey test (α = 0.05).

**Results:**

In irradiated roots, fracture resistance of EGCG and curcumin groups did not differ from non-instrumented roots, but they were higher than the NaOCl group (P = 0.006). However, NaOCl, EGCG, and curcumin in irradiated roots had comparable strength that was higher than in the non-instrumented group (p<0.001). Difference between irradiated and non-irradiated roots was observed only for NaOCl and non-instrumented groups (P≤0.004). In irradiated roots, a higher sealer penetration was observed in EGCG and curcumin groups compared to NaOCl.

**Conclusion:**

EGCG and curcumin could be promising final irrigants to reverse the adverse effect of radiotherapy on the strength of irradiated roots obturated with AH Plus sealer.

## Introduction

Radiotherapy alone and/or combined with surgery and chemotherapy is the treatment widely used in head and neck cancers [1,2]. Radiotherapy significantly increases cure rates; however, the high-energy ionizing radiation may lead to various adverse effects in the radiation field [2,3]. Radiation caries is the main complication in the oral cavity [4]. Dentition breakdown induced by radiotherapy initiates immediately following radiotherapy and rapidly progresses [3,5]. This damage occurs as a consequence of chemical and structural alterations of teeth particularly when accompanied by a decrease in the quantity and quality of saliva, poor oral hygiene, increased intake of cariogenic foods, and changes in the oral microbiota [6]. Together they all might lead to an increased risk of progression of rapid radiation caries over time [5,7,8]. Moreover, radiotherapy was reported to induce nuclear alterations in pulps [9]. These pulpal changes along with severe destruction of coronal structures enhance the need for endodontical treatments in radiotherapy-treated patients.

It is noteworthy that success in endodontic treatments faces some challenges for these patients. Several studies demonstrated the damaging effects of radiotherapy on the organic matrix of root dentin structure such as fragmentation and denaturation of collagen fibers and decreased root dentin stability [6,10,11]. These findings may help explain the observed significant reduction of bond strength of adhesive sealers to irradiated root canal dentin [12]. In addition, when radiotherapy and root canal treatment are accompanied together, the deleterious effects on dentin microhardness would be increased [13]. Although a decrease in the fracture resistance of teeth after radiotherapy has been reported [14]; However, no attempts have been made to reverse the adverse effects of radiotherapy on the strength of endodontically treated roots so far. Final irrigation with NaOCl and EDTA solutions is well-known as a standard protocol. However, this irrigation resulted in collagen degradation, demineralization, and peritubular and intratubular dentin erosion [15,16] revealed in an SEM analysis study [17]. OCl anions induce an acidification process leading to a mineral loss that causes a brittle matrix, reducing elastic modulus, microhardness, and fracture strength of dentin [18,19].

Epigallocatechin gallates (EGCG) as a polyphenol compound of green tea is extracted from the camellia plant and has antioxidant and anti-inflammatory properties [20]. EGCG is also known to promote collagen cross-linking, improving biomechanical properties and stabilization of collagen properties [21]. The use of EGCG as a final irrigant was reported to increase the bond strength of an epoxy resin sealer to root dentin [16]. Moreover, the effective antibacterial property of EGCG against Enterococcus Faecalis biofilm was documented. This effect occurs through hydroxyl radical generation [22,23].

Curcumin is another natural polyphenol, an active ingredient of turmeric, which is derived from the underground stems of Curcuma longa. It was recognized to have antimicrobial, antioxidant, and anti-inflammatory characteristics. Similar to ECGC, it was known as a polyhydroxide crosslinker [24]; hence both of them as compounds with minimal toxicity/side effects are attractive in various dentistry fields such as adhesive dentistry. The use of curcumin with or without photodynamic therapy was demonstrated as a suitable intracanal irrigant, providing effective disinfection and disrupting root canal biofilm [25,26]. However, contrary to EGCG, the effect of curcumin irrigant on the efficacy of adhesive sealers has not been addressed.

It is important to preserve irradiated teeth which may help maintain the quality of life in cancerous patients. Consequently, improving the root fracture resistance of irradiated teeth that need endodontic treatment is an important issue. To the extent of our knowledge, there are no studies regarding using crosslinking/antioxidant agents as a final irrigant in the irradiated root. Accordingly, this study aims to investigate the effect of two natural polyphenol crosslinking/antioxidant agents, as final irrigation solutions, on the root fracture resistance of irradiated endodontically-treated teeth and also to assess the sealer penetration in dentinal tubules by confocal laser scanning microscopy (CLSM). The null hypothesis is that the use of EGCG and curcumin would not affect the root fracture resistance and sealer penetration of irradiated and non-irradiated endodontically-treated teeth.

## Methods and materials

### Gathering, selection, and classification of specimens

The study protocol was approved by the Local Research and Ethics Committee (Protocol # IR.SUMS.DENTAL.REC.1400.080). Eighty mandibular premolars, with single and straight roots, which were extracted for orthodontic reasons (non-research purposes) were selected from 16 to 25 years old patients. Informed written consent was taken from all the participants whose teeth were used in the study. The teeth with cracks, caries, restoration, and calcification have been excluded. The teeth were cleaned and then kept in 0.5% chloramine solution at 4°C temperature. The teeth had similar sizes (±20% Mean) based on measuring buccolingual and mesiodistal dimensions at the cementoenamel junction (CEJ).

#### Radiotherapy procedure

The teeth were randomly divided into 8 groups (n = 10): four groups were not irradiated, and the other four groups were subjected to fractionated radiation. Forty teeth assigned to receive radiation were placed in plastic vials containing distilled water in a way to receive a uniform radiation dose. A total of 60 Gy irradiation consisting of 2 Gy exposure applied 5 days per week (for 6 weeks) with 6 MV X-rays from a linear accelerator (Vital Beam, Varian®, Palo Alto, CA, USA) was applied on the samples. During radiotherapy, the samples were submerged in distilled water which was changed daily. The beam geometry and the radiation dose were selected similarly to the beam setup and prescription dose of patients with head and neck tumors. Plans were performed using TPS software (Eclipse, version 15, Varian®). Also, the samples of the non-irradiated groups were stored in the distilled water which was renewed daily to mimic the condition similar to the irradiated groups.

### Preparation of specimens

The teeth were sectioned below the CEJ perpendicular to the long axis of the roots so that the crowns were removed and the remaining length of the roots was 13 ± 1 mm. Two control groups were kept non-instrumented including non-irradiated (group 1) and irradiated (group 2). The root canals of the other six groups (experimental groups) were instrumented with the ProTaper NiTi rotary system (Dentsply, Maillefer, Ballaigues, Switzerland) up to size F4. The working length of each sectioned root was considered to be 2 mm shorter than the root length. After using each file, 2.5% NaOCl (Sigma-Aldrich, St. Louis, MO, USA) via a 27-G irrigating tip (Endo-Eze; Ultradent, South Jordan, UT) was used for root canal irrigation. The outward flow of irrigant from the apices was prevented by sealing the apices of the roots with wax. Following the completion of root canal instrumentation, the experimental samples were irrigated with 5 mL 2.5% NaOCl and 2 mL saline. All the materials used in the study are represented in Table 1.

**Table 1:** Comparison of fracture resistance between groups.

	Non-irradiated	Radiated	P-Value
**Non-Inst**	1277.8±134.267 Aa	1010.2±107.380 Ba	<0.001
**NaOCl**	982.3±75.76 Ab	856.3±94.08 Bb	0.004
**EGCG**	1017.5±98.38 Bb	997±91.14 Ba	0.635
**Curcumin**	1037.6±115.2 Bb	1003.8±127.97 Ba	0.543
P-Value	<0.001	0.006	0.002

Different capital letters indicate a significant difference between rows analyzed by independent t-test, and lowercase letters indicate a significant difference between columns analyzed by Tukey’s test.

**Fig 1:**
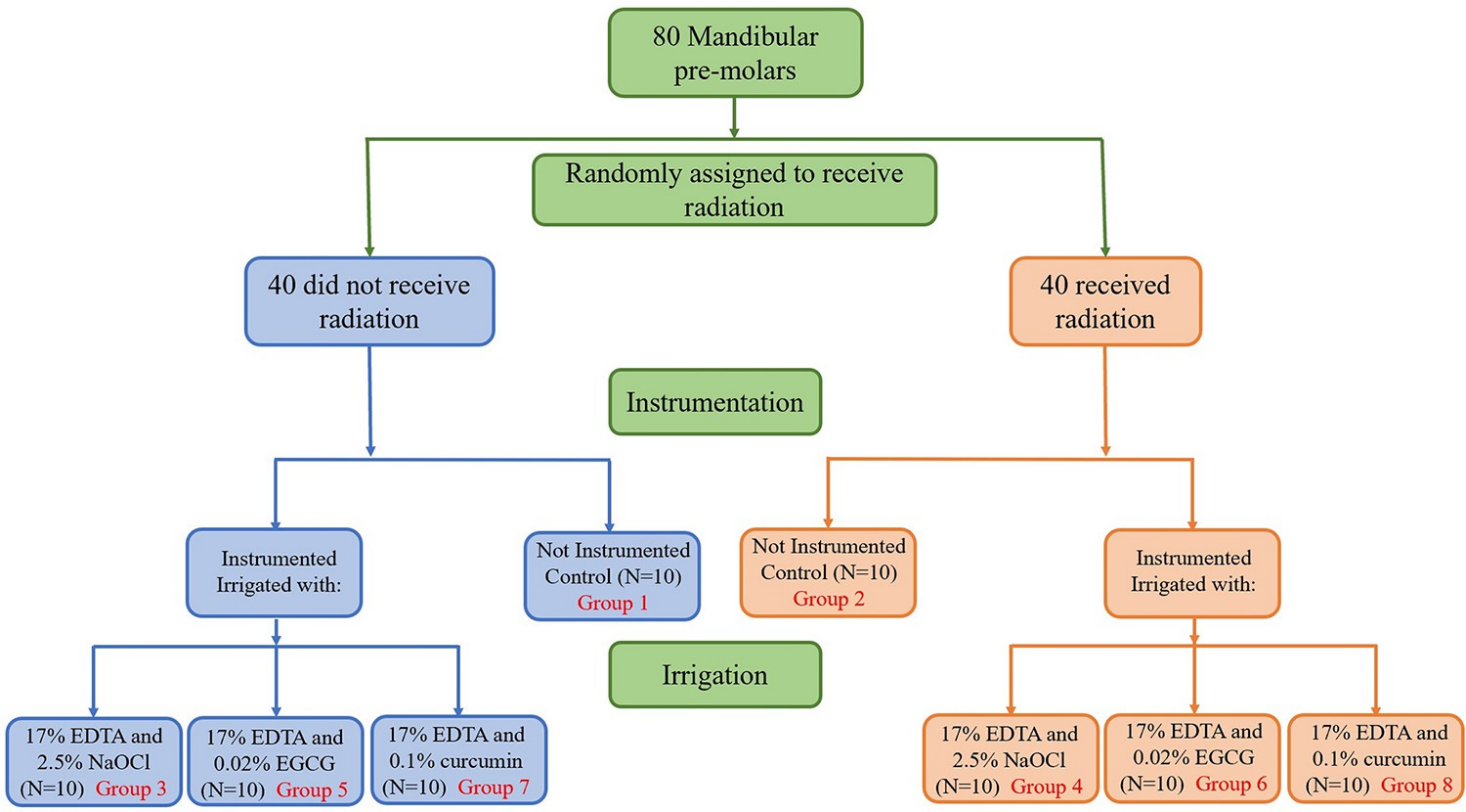
Flowchart of the study.

The 2 control and also 6 experimental groups based on irradiated/non-irradiated and final irrigants used including NaOCl, EGCG, and curcumin are as follows (The flowchart of the study is demonstrated in Fig 1):

1. Non-IR/Non-Instrumented
2. IR/Non-instrumented
3. Non-IR/EDTA + NaOCl: Non-irradiated root canal irrigated with 17% EDTA (5 mL, 1 min) and 2.5% NaOCl (5 mL, 1 min).
4. IR/EDTA+ NaOCl: Irradiated root canal irrigated with 17% EDTA (5 mL, 1 min) and 2.5% NaOCl (5 mL, 1 min).
5. Non-IR/EDTA + EGCG: Non-irradiated root canal irrigated with 17% EDTA (5 mL, 1 min) and 0.02% Epigallocatechin gallate aqueous solution (5 mL, 1 min).
6. IR/EDTA+ EGCG: Irradiated root canal irrigated with 17% EDTA (5 mL, 1 min) and 0.02% Epigallocatechin gallate (5 mL, 1 min).
7. Non-IR/EDTA + curcumin: Non-irradiated root canal irrigated with 17% EDTA (5 mL, 1 min) and 0.1% curcumin solution in 1% DMSO (5 mL, 1 min).
8. IR/EDTA+ curcumin: Irradiated root canal irrigated with 17% EDTA (5 mL, 1 min) and 0.1% curcumin in 1% DMSO (5 mL, 1 min).

Each root canal was rinsed with 5 mL of distilled water to remove any residual irrigant solution after final irrigation. After drying the root canals with paper points, the roots were filled with ProTaper F4 gutta-percha (Dentsply Maillefer, Ballaigues, Switzerland) and AH Plus root canal sealer (Dentsply DeTrey, Konstanz, Germany) using a single-cone technique.

After root filling, the coronal 1 mm of the filling materials was removed with a carrier and condensed using a hand plugger, and the spaces were filled with a temporary filling material (Cavit; 3M ESPE, Seefeld, Germany). The adequate obturation quality of the root canal fillings was confirmed using mesiodistal and buccolingual periapical radiographs. All samples were placed in 100% humidity and at 37°C for 24 hours to allow the sealer setting [27].

### Preparation for fracture resistance test

Samples were vertically mounted in self-curing acrylic resin (Acropars, Marlik Co., Tehran, Iran) block up to 2 mm below the CEJ. To mimic the periodontal ligament, the root surfaces were covered with a 0.2–0.3 thick layer of silicone material (C-silicone, Zetalabor, Zhermack SpA, Badia Polesine, Italy). After attaching the acrylic blocks to the lower plate of a universal testing machine (Z020, Zwick Roell, Ulm, Germany), a steel ball with a spherical tip (5 mm in diameter) was mounted on the upper plate and was placed over the center of the root canal orifice to apply an increasing compressive load at a speed of 1 mm/min to the root parallel to its long axis until fracture. A sudden drop in the recorded forces indicates an occurrence of a crack initiating fracture of the root. The maximum force required to fracture each root was recorded in Newtons (N). After removing it from the acrylic mold, the occurrence of the fracture was confirmed using a 4× magnifying glass [28].

### Confocal laser scanning microscope analysis

Two additional root specimens from the six experimental groups were prepared to evaluate the sealer penetration by confocal laser scanning microscopy (CLSM). All preparation steps were in the same manner as mentioned for each respective group in the FR test except that the sealers were mixed with rhodamine B (Sigma-Aldrich, St. Louis, Mo, USA), as a fluorescent marker, at a 100:1 ratio by weight [29]. Each root specimen was sectioned perpendicular to its long axis using a precision saw (IsoMet 1000; Buehler, Lake Bluff, IL) at a slow speed under water cooling. Three slices were obtained from the middle third with approximately 1mm in thickness. The sections were polished with silicon carbide abrasive papers and then ultrasonically cleaned for 15 minutes to remove residual debris. The samples were then mounted onto glass slides and examined with a Leica TCS-SPE confocal laser scanning microscope (Leica, Mannheim, Germany) at ×10 with a wavelength of 550–650 nm. Digital images were provided with the Leica LAS X software. In case the entire canal could not be examined in one image, further partial images were taken and then assembled as a single image using Photoshop (Adobe Systems, Inc., San Jose, CA).

### Data analysis

The normality of the data was confirmed using the Kolmogorov–Smirnov test. The Two-way ANOVA followed by the Tukey post hoc test was used for comparing the values of FR and dye penetration assessed by CLSM between the 8 groups. Independent sample T-test was used to compare the values between irradiated and non-irradiated groups for each type of irrigation. All analyses were conducted using SPSS software version 17 (SPSS Inc, Chicago, USA). The statistical comparisons were considered significant at *P* values less than 0.05.

## Results

The results of the fracture resistance are displayed in Table 1. Two-way ANOVA revealed a significant effect for irradiation (P<0.001), final irrigant (P<0.001), and interaction between irradiation and irrigant (P = 0.002). In non-irradiated groups, non-instrumented roots had significantly higher fracture resistance than NaOCl, EGCG, and curcumin groups (P<0.001) which had comparable values. In irradiated roots, non-instrumented, EGCG, and curcumin groups had comparable fracture resistance that was significantly higher than the NaOCl group (P = 0.006).

The fracture resistance of non-instrumented and NaOCl groups of irradiated roots was significantly lower than non-irradiated ones (P<0.001 and P = 0.004). This comparison revealed no difference for EGCG and curcumin groups (P>0.05).

**Fig 2:**
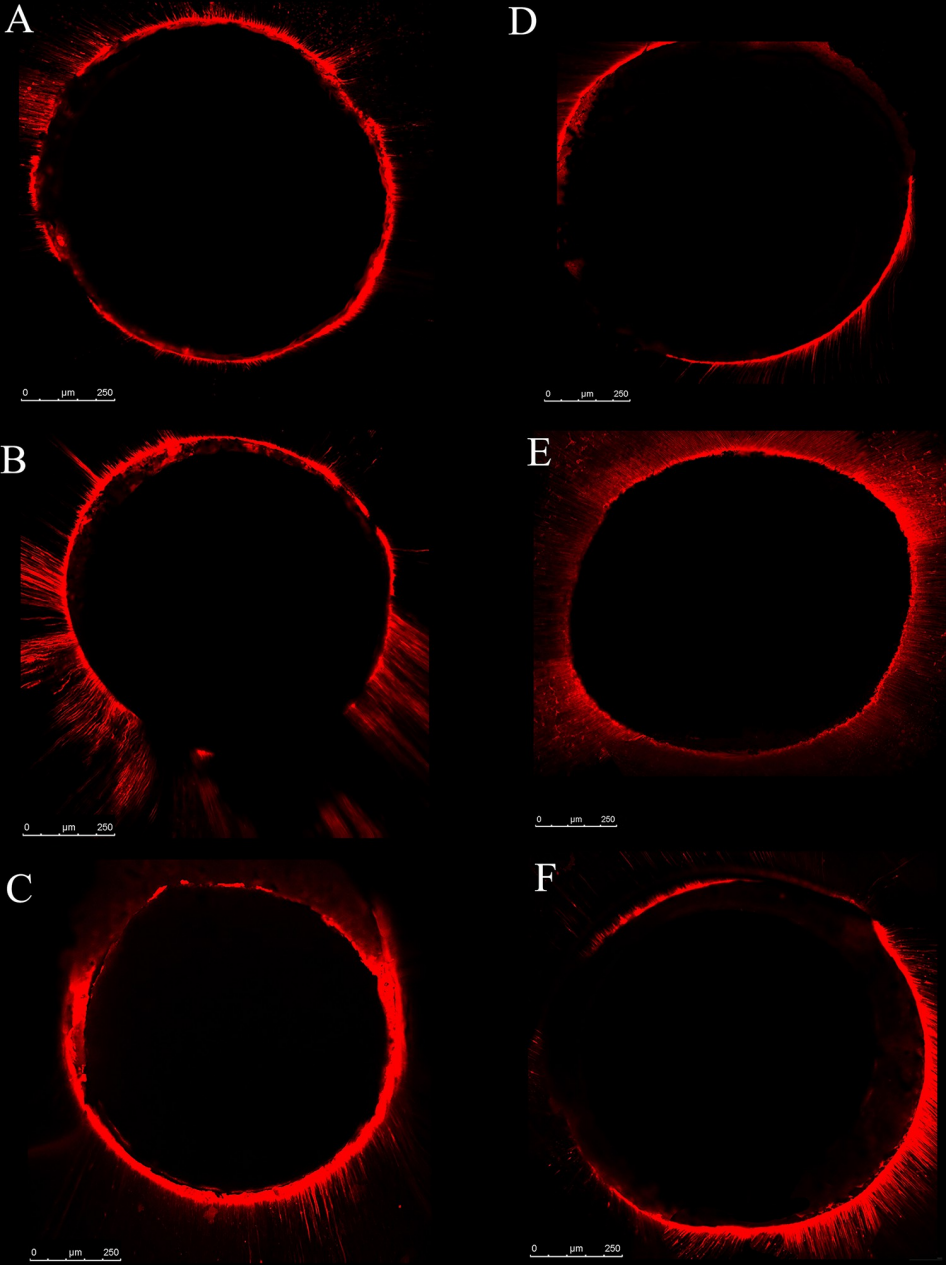
Confocal laser scanning micrographs (CLSM) displaying sealer penetration in the groups with different irrigants. **A)** Non-irradiated teeth treated by NaOCl as final irrigants, **B)** Non-irradiated teeth treated by EGCG as final irrigants, **C)** Non-irradiated teeth treated by Curcumin as final irrigants, **D)** Irradiated teeth treated by NaOCl as final irrigants, **E)** Irradiated teeth treated by EGCG as final irrigants, **F)** Irradiated teeth treated by Curcumin as final irrigants.

Confocal laser scanning micrographs (CLSM) displaying sealer penetration in the groups with different irrigants are presented in Fig 2. The dye penetration values in μm are presented in Table 2. Confocal images showed a relatively uniform fluorescent layer at the interface with root dentin and a consistent penetration in the dentinal tubules in both irradiated and non-irradiated specimens. In non-irradiated roots, no difference in penetration depth was observed according to the type of irrigants (P = 0.360). Nevertheless, in irradiated roots, EGCG and curcumin groups displayed significantly higher penetration than the NaOCl group (P = 0.032, P = 0.023 respectively).

**Table 2:** Mean ± standard deviation of dentinal tubule penetration depth (μm).

	Non-irradiated	Radiated	P-Value
**NaOCl**	240.8±129.09 Aa	177.94±44.55 Bb	0.002
**EGCG**	312.53±103.45 Aa	287.18±58.17 Aa	0.191
**Curcumin**	262.59±53.02 Aa	245.27±61.88 Aa	0.978
P-Value*	0.360	0.003	

* Analyzed by One-way ANOVA.

Different capital letters indicate a significant difference between rows analyzed by independent t-test and lowercase letters indicate a significant difference between columns analyzed by Tukey’s test.

## Discussion

A lower fracture resistance of the EDTA/NaOCl irradiated group compared to the non-irradiated group was observed in the present study and based on these results, using EGCG and curcumin irrigant instead of NaOCl as final irrigants could significantly restore the negative effect of radiotherapy on root fracture resistance. However, the non-irradiated root did not benefit from any of the two cross-linkers used. Therefore, the tested null hypothesis was partly rejected.

The mentioned findings may be indirectly related to the lowered bonding efficacy of AH Plus sealer, or it could be directly explained by the weakening and damaging effect of radiotherapy on the protein component of the root canal dentin, and or a combination of both. Soares et al. have previously reported decreased fracture resistance and increased cuspal strain of gamma-irradiated endodontically treated maxillary premolars. They suggested that the protein degradation and weaker interprismatic substance in irradiated specimens vs non–irradiated ones induce a rapid stress propagation, thereby fractures occurring under a lower load [14]. Contrary to our result, a recent study displayed that when radiotherapy had been conducted before endodontic treatment, it did not affect fracture resistance of premolars obturated with Biodentine or gutta-percha/epoxy resin-based sealer [30]. The difference in methodologies, irradiation source, experimental design, and in vitro conditions can contribute to the different findings reported. Radiotherapy-induced strength loss was reported in the case of intact teeth. This enhanced susceptibility to fracture after radiotherapy was attributed to decreased hardness and elasticity of tooth structures [31].

Radiation can damage the dentin collagen network by scission of collagen peptide bonds [6]. Radiolysis, the interaction between radiotherapy and the water content of dentin, leads to the production of free radicals and hydrogen peroxide. The released ions induce an alteration in the chemical and ultrastructure of dentin [32]. This alteration may contribute to crack formation, obliteration of dentinal tubules, dryness, and fragility of irradiated dentin. These factors along with altered secondary and tertiary structures of proteins may be responsible for less adhesive receptiveness of the dentin [31,32]. Accordingly, radiotherapy could interfere with the function of the resin sealer.

Endodontic sealers establish intimate contact and penetration into the dentinal tubules and consequently, a perfect seal against recontamination is achieved [33]. AH Plus is used in the present study as an adhesive endodontic sealer. This hydrophobic epoxy resin sealer can bond chemically to the exposed amino groups of collagen fibers of root canal dentin [34]. As it was explained, the damaged organic part of the irradiated root dentin might prevent the formation of a strong bond to the resin sealer. Despite the controversial reports [35,36], some authors have demonstrated that effective bonding of adhesive materials to the root dentin may provide a strengthening effect [37–40]. This can be a possible explanation for a considerably lower FR of NaOCl–irrigated root in the irradiated group compared to the non-irradiated group. The FR results were corroborated with confocal microscopy observation in the current study so that the depth of sealer penetration in the irradiated samples was significantly lower than in the non-irradiated ones. In line with our results, decreased bond strength due to radiation and more gaps at the sealer-dentin interface were reported in another study [12].

The final irrigation regiments have a major role in the success of endodontic treatment [37]. Removal of the smear layer using irrigants and usage of surfactants in final irrigants enhance the contact and penetration of sealers into dentinal tubules. This may increase the root strength [41]. Despite these desirable properties, they should not adversely influence the mechanical strength and quality of root canal structure; in particular, in the case of irradiated root canal dentin with reduced stiffness and modulus of elasticity [31]. The conventional final irrigant used in our study was based on the most effective regimen in creating a smear layer-free surface (17% EDTA for 1 minute followed by 2.5% NaOCl) [27,42,43]. However, this protocol may decrease the bond strength of epoxy resin sealers caused by collagen degradation [44,45]. This degradation could be exaggerated in irradiated root dentin. Also, it was demonstrated that the prolonged EDTA application could cause dentin erosion and an increased risk of root fracture [46]. Therefore, final flushing with a collagen stabilizer/antibacterial agent, especially in damaged irradiated root dentin could be considered in the endodontic treatment procedure of irradiated teeth. Furthermore, higher resistance to vertical root fracture was obtained when chlorhexidine rinsing was used after EDTA/NaOCl as a final irrigant [27].

A different effect of crosslinking agents on irradiated and non-irradiated roots was observed in our study. In the 3 irradiated groups, both EGCG and curcumin irrigants compared to NaOCl could provide a significantly higher depth of sealer penetration and fracture resistance. It has been shown that radiotherapy imposes a damaging effect on the collagen matrix of the root dentin which interferes with the bonding of AH Plus sealer to collagen fibers. It is possible that the cross-linking efficacy of EGCG and curcumin could restructure/re-stabilize damaged collagens [21,47] increasing the mechanical strength of weakened irradiated root dentin. In addition, it was reported curcumin increased the mechanical properties of intraradicular dentin [48]. Similarly, EGCG may help the establishment of hydrogen bonding and hydrophobic interactions in collagen molecules through its hydroxyl and carboxyl groups [49]. The interaction of curcumin with collagen consists of hydrogen bonding and strong electrostatic charge interaction [50].

Radiotherapy activates matrix metalloproteinase enzymes (MMP) and also produces free radicals [51]. Both Curcumin and especially EGCG have strong antioxidant properties which may reverse the effect of free-radical produced in radiotherapy [20,24]. Moreover, EGCG can inhibit MMPs and cysteine-cathepsins; hence improving adhesive interface durability [52]. Similarly, curcumin has been shown to increase the degradation resistance of dentin collagen matrix and improve resin dentin bond strength after aging [53] which was attributed to its inhibitory effect on MMPs [50]. This anti-MMP property may explain the observed effects of the dentin bio-modifying agents used in this study in irradiated groups.

Previously, the positive effect of carbodiimide (EDC) as a synthetic cross-linker in the restructuring of the secondary structure of the RT-induced damaged dentin and restoring collagen integrity was reported [10]. In the irradiated root, EDC was reported to improve bonding longevity as a self-adhesive resin for the cementation of fiber posts [54]. In the study by Pheenithicharoenkul et al, an improved bond strength of AH Plus to normal root dentin following applying EGCG as the final irrigation was reported [16]. However, in our study, the non-irradiated group’s FR was unaffected by EGCG. In Pheenithicharoenkul’s study, EDTA was applied for 5 minutes, leading to collagen collapse. Consequently, EGCG helped to stabilize collagen fibers. However, In our study, EDTA was used only for 1 minute considering the chelating capability of EGCG which can compensate for the shorter application time of EDTA. This might be an explanation for the numerically better penetration of the sealer observed in confocal images of the two EGCG irrigated groups in our study.

The present study faces some limitations as it is performed in a static condition. The fracture resistance of sectioned roots was measured by applying a compressive axial load over the canal orifice. This experiment setup could provide a standardized condition [27] which has been used in a number of studies to assess the effect of a different endodontic intervention such as final irrigation [27,28,41,55]. The fracture resistance of the obturated root is dependent on several factors in the clinical situation such as residual coronal structure, type of coronal restoration, use of intra-canal post and core type [55]. Hence it can be expected that a static loading test, similar to what is used in this study, cannot mimic clinical intra-oral functional loading. In addition, they reveal a fracture resistance value that is not similar to those from clinical situations [56]. However, the present method is still acceptable as it can omit other interfering conditions and can compare the effect of a single factor such as different irrigants on root resistance to vertical fracture. Dynamic fatigue loading or a combination of mechanical loading with thermal cycling can provide a more relevant situation to clinical oral conditions. Also, finite element analysis evaluation would reveal significant information about biomechanical performance at endodontically treated teeth. Finally, clinical evidence is required to validate these experimental findings. Further studies are suggested to assess the effect of the other antibacterial agents such as chlorhexidine and other crosslinkers in different concentrations and application times and with various adhesive resin sealers on the strength of the irradiated root.

## Conclusion

Radiotherapy before endodontic treatment adversely affected the root fracture resistance and penetration depth of epoxy resin sealer. Using EGCG and curcumin as final flushing could be suggested to reverse this effect, improving the resistance of the irradiated root to vertical loading and fracture.
